# Supplementary material for: Development and Validation of the Interpersonal Motivational Systems Questionnaire (IMS-Q)
Source: Behav Sci (Basel). 2023 Sep 21;13(9):784. doi: 10.3390/bs13090784 (PMC10525080; doi:10.3390/bs13090784)
Supplement: Supplementary file 1 [file behavsci-13-00784-s001.zip › behavsci-2542270-supplementary.pdf]

Supplemental material

IMS-Q (English version)

The following statements pertain to your own attitudes, emotions, and actions. Please read each statement and evaluate how closely it aligns with your usual feelings and behaviors across various life domains, such as work, family, and more.

You can respond to each statement by selecting from the options provided.

**0**	**1**	**2**	**3**
Never	Rarely	Sometimes	Often

Please respond to each sentence, ensuring that you address every one of them. If a question pertains to your partner, consider how you typically behave in an emotional relationship. Remember that you are providing your own self-assessment, and there are no right or wrong answers.

	**0**	**1**	**2**	**3**
1. I happen to feel inferior to others				
2. When I'm with my colleagues/collaborators/friends, I feel as if we share the same goals				
3. I happen to help others because I feel they need it				
4. I happen to feel equal to others, on the same plane of dignity				
5. I happen to tell playful stories to play it down, but without ridiculing the other person				
6. When I compare my interests with those of the members of my group (e.g., colleagues, friends, collaborators, and other groups), I can see some affinities				
7. I find myself imaging sexual scenes with real and/or imaginary partners that excite me				
8. I happen to feel my partner close to me when I need it				
9. I like to feel having equal dignity while interacting with another person				
10. I happen to think that people are less able than me				
11. I happen to feel the urge to take care of others and their needs				
12. I happen to make seductive compliments				
13. I happen to avoid competition for fear of receiving a negative judgment from others				
14. I happen to take care of others, though they don't ask for it				
15. I find myself making jokes that others enjoy, without offending anyone				
16. I happen to give permissions and prohibitions to others				
17. When someone shouts at me, I happen to look down				
18. I like to tell fun stories/jokes just for fun purposes				
19. I feel like I'm equal in dignity to others, even though I'm helping them				
20. I happen to believe my partner shows me the love I deserve				
21. I prefer to have someone to guide me and tell me what to do when facing important decisions				

22. I happen to be sexually attracted to others				
23. I happen to provide others with comfort, assurance, and protection				
24. I happen to be playful and friendly with others				
25. I like to feel self-satisfied with my successes and show others my victories				
26. I feel like I'm equal in dignity to others, even though I'm asking them for help				
27. I happen to feel my partner supports me when I need it				
28. When I'm with my friends, I happen to feel like part of the group				
29. I happen to go out to seduce or sexually provoke people				
30. I happen to trust my partner				
31. When someone verbally takes me back, I feel humiliated/scorned				
32. When I partake in activities with the members of a group, I happen to feel the pleasure of doing it				
33. I find myself worrying about others and the risks they can take				
34. I happen to take control of situations				
35. I happen to think that I'm on the same plane as another person, independently of our social roles				
36. I happen to use the terms "us" or "we" to refer to my group (e.g., class, team, fellow citizens, clubs, friends, and other groups)				
37. I happen to tell others about my sexual attractions, without any shame				
38. I happen to organize funny jokes, but not hurtful				
39. I worry and get active if someone is in danger				
40. I happen to give up easily in disputes				
41. I happen to verbally frighten others				
42. I happen to think I have interchangeable roles with others, since, as humans, we have the same dignity				
43. I happen to feel like my partner won't leave me				
44. I happen to accept, without embarrassment, erotic compliments				
45. I happen to alter my voice to have fun with others (e.g., I change my voice, I do strange or funny voices, etc.)				
46. I happen to feel like I'm part of a wider group (e.g., "we women," "we men," "we young people," "we elders," etc.)				
47. I happen to be seduced by seductive attitudes or ways of dressing				
48. I happen to get really ashamed when I make a fool of myself				
49. I can propose funny games/activities to others, without imposing myself				

50. I happen to notice that people look at me seductively				

IMS-Q (Italian version)

Le frasi che seguono riguardano atteggiamenti, sentimenti e comportamenti relativi a se stesso/a. Legga ogni frase e decida quanto ciascuna corrisponde al suo carattere, riferendosi a come abitualmente si sente nella sua vita e nei diversi contesti (es. lavoro, famiglia, ecc.).

Per ciascuna affermazione, può rispondere scegliendo tra:

**0**	**1**	**2**	**3**
Mai	Raramente	Abbastanza	Spesso

Risponda comunque a ciascuna frase, senza tralasciarne alcuna; in presenza di domande riferite al partner si riferisca a come abitualmente si pone in una relazione affettiva. Ricordi di dare la sua opinione su di sé, non ci sono risposte giuste o sbagliate.

	**0**	**1**	**2**	**3**
1. Mi capita di sentirmi inferiore rispetto agli altri				
2. Quando sono con colleghi/soci/amici sento di condividere gli stessi obiettivi				
3. Mi capita di aiutare gli altri perché sento che ne hanno bisogno				
4. Succede di sentirmi alla pari con gli altri, sullo stesso piano in quanto a dignità				
5. Mi capita di raccontare storie giocose per sdrammatizzare ma senza svalutare l'interlocutore				
6. Quando confronto i miei interessi con quelli dei membri del gruppo di cui faccio parte (es. colleghi, amici, soci, comitive, ecc.) mi capita di trovare affinità				
7. Mi ritrovo ad immaginare scene sessuali con partner reali e/o immaginari che mi eccitano				
8. Mi capita, se ho bisogno, di sentire il mio partner vicino				
9. Provo piacere nel sentirmi alla pari dignità con un altro quando interagisco				
10. Mi capita di pensare che gli altri siano meno capaci di me				
11. Mi capita di sentirmi spinto a prendermi cura dell'altro e dei suoi bisogni				
12. Mi succede di fare complimenti seduttivi				
13. Mi capita di evitare le competizioni per paura di ricevere un giudizio negativo dagli altri				
14. Mi capita di prendermi cura degli altri anche se non mi è richiesto				
15. Mi ritrovo a fare battute che divertono gli altri senza offendere nessuno				
16. Mi capita di dare permessi o divieti agli altri				
17. Quando qualcuno alza la voce mi succede di abbassare lo sguardo				
18. Mi capita di raccontare storie divertenti/barzellette con intento giocoso				
19. Mi sento di pari dignità con l'altro anche se lo sto aiutando				
20. Mi succede di pensare che il mio partner mi dia l'affetto che merito				

21. Preferisco avere qualcuno che mi guidi e mi dica cosa fare di fronte a decisioni importanti				
22. Mi succede di essere attratto sessualmente dagli altri				
23. Mi succede di dare conforto rassicurazione e protezione agli altri				
24. Mi capita di essere scherzoso e giocoso con gli altri				
25. Mi piace autocompiacermi dei miei successi ed evidenziare agli altri le mie vittorie				
26. Mi sento di pari dignità con l'altro anche se gli chiedo aiuto				
27. Mi capita, se necessario, di sentirmi confortato dal mio partner				
28. Quando sono con i miei amici mi capita di sentirmi parte integrante del gruppo				
29. Mi succede di uscire con l'intento di sedurre o provocare sessualmente				
30. Mi capita di fidarmi del mio partner				
31. Quando mi riprendono verbalmente mi sento umiliato/disprezzato				
32. Quando svolgo attività condivise con i membri di un gruppo mi capita di provare il piacere di partecipare				
33. Mi ritrovo a preoccuparmi per gli altri per i pericoli che possono correre				
34. Mi capita di prendere il comando delle situazioni				
35. Mi capita di pensare che al di là dei ruoli sociali io e l'altro siamo sullo stesso piano				
36. Mi ritrovo ad utilizzare il termine "noi" riferendomi al mio gruppo di appartenenza (es. classe, squadra, italiani, club, amici, comitiva, ecc.)				
37. Mi capita di raccontare agli altri delle mie attrazioni sessuali senza vergogna				
38. Mi capita di organizzare scherzi divertenti, non offensivi				
39. Mi preoccupo e mi attivo se qualcuno è in pericolo				
40. Mi capita di arrendermi facilmente nelle dispute				
41. Mi capita di intimorire l'altro verbalmente				
42. Mi capita di pensare di avere ruoli intercambiabili con l'altro in quanto persone di pari dignità				
43. Mi capita di sentire che il mio partner non mi abbandonerà				
44. Mi capita di accettare, senza imbarazzo, i complimenti a sfondo erotico				
45. Mi capita di "caratterizzare" la mia voce per scherzare con gli altri (es. cambio voce, fare voci strane e divertenti, ecc.)				
46. Mi succede di sentirmi parte di un gruppo più ampio (es. "noi donne", "noi uomini", "noi giovani", "noi anziani", ecc.)				
47. Mi succede di essere sedotto da atteggiamenti o dal modo di vestire seducente				
48. Mi capita di vergognarmi fortemente quando faccio una brutta figura				

49. Mi ritrovo a proporre giochi/attività divertenti ad altri senza impormi				
50. Mi capita di notare che le persone mi guardano in modo seducente				

Scoring system

The IMS are different motivational systems that guide our social interactions, and each one consists of a set of items that assess its underlying motivational tendencies.

To compute a score for each of the eight IMS (Interpersonal Motivational Systems) for an individual, average the items that index it, as listed in the table below.

System	Item number
Accudimento (Caregiving)	3,11,14,23,33,39
Affiliazione (Social affiliation)	2,6,28,32,36,46
Attaccamento (Attachment)	8,20,27,30,43
Dominanza (Dominance)	10,16,25,34,41
Gioco (Social play)	5,15,18,24,38,45,49
Cooperazione (Cooperation)	4,9,19,26,35,42
Sessualità (Sexuality)	7,12,22,29,37,44,47,50
Sottomissione (Submission)	1,13,17,21,31,40,48
